# A Difficult Recurrent Hypertrophic Pyloric Stenosis

**DOI:** 10.21699/jns.v6i2.484

**Published:** 2017-04-15

**Authors:** Hamdi Louati, Hayet Zitouni, Manel Belhajmansour, Mahdi Ben Dhaou, Mohamed Jalouli, Riadh Mhiri, Rim Kallel, Tahya Boudawara

**Affiliations:** 1 Department of Pediatric Surgery, Hedi Chaker Hospital, 30219, Sfax, Tunisia; 2 Department of Anatomopathology, Habib Bourguiba Hospital, 30219, Sfax Tunisia

**DEAR SIR**

A 20-day-old girl presented with increasing projectile vomiting over a period of one week and progressive weight loss. The diagnosis of hypertrophic pyloric stenosis (HPS) was made by sonography (Length of pyloric channel: 22 mm; muscle thickness: 5 mm). Open standard pyloromyotomy was performed. The postoperative course uneventful. On the 50th postoperative day, the girl was readmitted with projectile vomiting for a week. Contrast radiography proved a pyloric stenosis occlusive. Ultrasound confirmed recurrent pyloric stenosis (length of pyloric channel: 22 mm; thickness of the muscle: 4.8mm). At repeat surgery, the previous incision had been healed (Fig.1). A pyloromyotomy was done on lateral side. The postoperative period was complicated by vomiting during each attempt of feeding. After 30 days of persistence of vomiting, patient was again explored and found completely healed pyloromyotomy incision. A pyloromyotomy with removing a piece of pyloric muscle from the edge of the myotomy was done and the histological examination show muscle cells hypertrophic. The postoperative course remained uneventful this time. The oral feeding was started on second postoperative day. The girl was discharged on fourth postoperative day. The baby is doing fine on follow-up. 

Immediate postoperative emesis following pyloromyotomy is not unusual and attributed to pyloric edema, gastroparesis, pylorospasm, or gastroesophageal reflux. However, emesis should resolve within days of surgery [1-4]. True recurrence of HPS is quite rare, defined as complete resolution of symptoms with subsequent weight gain followed by representation with sonographic and/or operative evidence of re-stenosis. The need of reoperation after pyloromyotomy because of recurrent vomiting is reported with an incidence up to 4% [3]. Heurn et al supported that if the first operation was done in the early stage of the developing disease it can be a cause of recurrent [3]. Khoshoo and colleagues [4] speculated that the failure of pyloromyotomy is more likely due to an incomplete pyloromyotomy at the gastric end. The underlying cause of a recurrent hypertrophic pyloric stenosis after a successful pyloromyotomy remains unclear but may be explained by the natural evolution of the disease [4]. Patients with recurrent vomiting after a successful pyloromyotomy and who have sonographic, and radiologic confirmation of recurrence should have an operative exploration [2]. Removing a piece of muscle throughout length of pyloromyotomy incision worked well in our case.

## Footnotes

**Source of Support:** None

**Conflict of Interest:** None

## Figures and Tables

**Figure 1:**
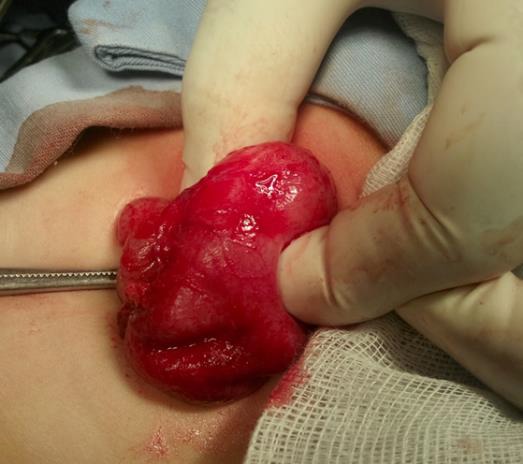
Operative view (in the second pyloromyotomy) showing a regular scar along the complete length of the pyloric muscle on the anterior side.
